# Direct evidence for grain boundary motion as the dominant restoration mechanism in the steady-state regime of extremely cold-rolled copper

**DOI:** 10.1016/j.actamat.2014.06.010

**Published:** 2014-09-15

**Authors:** O. Renk, A. Hohenwarter, S. Wurster, R. Pippan

**Affiliations:** a Erich Schmid Institute of Materials Science, Jahnstrasse 12, 8700 Leoben, Austria; b Department of Materials Physics, Montanuniversität Leoben, Jahnstrasse 12, 8700 Leoben, Austria

**Keywords:** Severe plastic deformation (SPD), Cold-rolling, Copper, Grain boundary migration, Steady state

## Abstract

Ultra-fine-grained high-purity copper (99.99%) deformed by means of high-pressure torsion into the steady-state regime was subjected to additional rolling deformation. The microstructural changes as a function of the applied strain were analysed by means of orientation imaging microscopy. It was found that after a distinctive rolling strain a steady state with respect to microstructural features such as grain size, misorientation distribution and texture evolves again. A special spilt specimen technique was used to perform quasi in situ observations of the microstructure between additional strain increments. Profound insights into the local deformation and restoration processes within the steady-state regime were gained. The observations lead to the conclusion that grain boundary migration perpendicular to the rolling direction leads to the disappearance of certain grains, enabling the occurrence of a steady state.

## 1 Introduction

In the last decades, severe plastic deformation (SPD) methods have made it possible to easily apply ultra-high strains to metals at low homologous temperatures. The applied strain leads to enormous grain refinement, resulting in ultra-fine-grained (UFG) or nanocrystalline (nc) materials. However, grain refinement is not indefinite and terminates after a certain amount of strain and the processed materials reach a steady state in deformation regarding microstructural features such as grain size, grain shape or misorientation distribution [1–9]. Additionally, defect densities such as vacancies or dislocations also remain constant. This steady-state grain size represents a lower limit for grain fragmentation as additional straining does not lead to further refinement. The occurrence of a distinct minimum grain size for a specific deformation technique at a given temperature and strain rate is confirmed by deforming a material with a starting grain size smaller than the steady-state grain size obtained by a certain SPD process. By doing this, the metal coarsens towards the steady-state size obtained by deforming a coarse-grained starting material [10]. In contrast to the starting grain size, deformation temperature, amount of impurities and strain rate influence the resulting steady-state grain size [1,2].

In the steady-state regime, restoration mechanisms have to take place at the same time in order to keep grain features constant. Estimating the minimum grain size after a certain SPD process is truly a challenging task; however, several attempts with contradicting assumptions have been recently made to predict or model the steady-state grain size [11–15]. In Ref. [11] it was suggested that the steady state is a consequence of the equilibrium between grain growth due to adiabatic heating and the refinement caused by straining. Divinski et al. [12] proposed that cracks initiate and propagate during SPD and limit the minimum grain size achievable. If crack initiation can be avoided during deformation, the steady-state grain size will be larger than the one calculated by their model. Edalati and Horita developed a model for predicting the steady-state hardness of pure metals after SPD as a function of their atomic bond energy and related parameters [13]. Another model proposed that the minimum grain size is a consequence of recovery and generation of dislocations, with the stacking fault energy being one of the most important parameters for the obtainable steady-state grain size [14,15]. In Ref. [1] it was suggested that the mobility of the grain boundaries should be the crucial parameter for the occurrence of a minimum grain size during SPD and larger grains become subdivided again by the formation of low-angle grain boundaries (LAGBs).

Despite the vast amount of scientific research in the field of SPD in the last decades and the above-mentioned recently published models for predicting the minimum grain size, the actual deformation and restoration mechanisms within the steady state have not been investigated directly in an experiment due to the obvious experimental difficulties one would have to face. In this contribution these difficulties were overcome by using an innovative experimental technique. The model material, UFG copper, was pre-deformed via high-pressure torsion (HPT) into the saturation regime and deformed further through rolling. The characteristics of rolling allowed for the use of a split specimen and to study quasi in situ the processes during the steady state, applying for SPD standards very moderate strain increments. The information gained from these experiments will give valuable input for further modelling activities.

## 2 Experimental

UFG copper with an area-weighted grain size of 530 nm was produced by high-pressure torsion. For that, discs of pure copper (99.99%) with a diameter of 30 mm and a height of 6.5 mm were processed with an applied pressure of 3.5 GPa for 15 revolutions, with a rotational speed of 0.07 rotations min^−1^. This results in a strain of *ε* = 92.1 at a radius of *r* = 11 mm according to Eq. (1) with the equivalent plastic strain *ε*, *n* the number of revolutions, *r* the radius and *t* the specimen thickness:

(1) $$\varepsilon =\frac{2\pi \mathrm{nr}}{t\sqrt{3}}$$

Details concerning the setup of the HPT tool used in this study can be found elsewhere [16]. Microhardness measurements along the radius of the copper disc were carried out to ensure that mechanically homogeneous properties were found across the entire disc, except for the very centre, *r* < 0.5 mm. The UFG copper discs were subsequently cold-rolled to different thickness reductions in a conventional rolling mill. The strain rate was kept fairly low. As a consequence, only small incremental thickness reductions and low rolling speeds were allowed in order to minimize self-heating of the copper sheet. The strain rate was estimated from Eq. (2) [17] to be $\overset{˙}{\varepsilon }=10^{-1}\;{\text{s}}^{-1}$, where Δ*t* is the difference in thickness before and after a rolling pass, *R* is the radius of the rolls, *ω* is the angular speed of the rolls and *ϕ* is the logarithmic thickness reduction:

(2) $$\overset{˙}{\varepsilon }=\frac{2}{\sqrt{3}}\frac{\omega \phi }{\sqrt{\frac{\Delta t}{R}}}$$

The applied rolling strain *ε* (equivalent strain) was calculated according to Eq. (3), with *t*_1_ the thickness of the copper sheet after cold-rolling and, *t*_0_ the thickness of the as-processed HPT disc (6.5 mm):

(3) $$\varepsilon =\frac{2}{\sqrt{3}}\ln\left(\frac{t_{1}}{t_{0}}\right)=\frac{2}{\sqrt{3}}\phi$$

Microstructures after HPT processing and after different amounts of additional cold-rolling were captured from electron backscatter diffraction (EBSD) data obtained with a Zeiss LEO 1525 field emission gun scanning electron microscope (SEM). Grain size was calculated from these data using an orientation imaging microscopy (OIM) analysis software package. About 2000 grains were analysed for each condition. Crystallographic texture of the samples was characterized using a Rigaku SmartLab five-axis diffractometer equipped with Cu Kα radiation, a parabolic multilayer mirror in the primary beam and a secondary graphite monochromator. The measurements were performed using a Schultz reflection technique. The collected pole figure data were evaluated using the software package Labotex™ in order to quantify volume fraction of individual texture components.

**Fig. 1:**
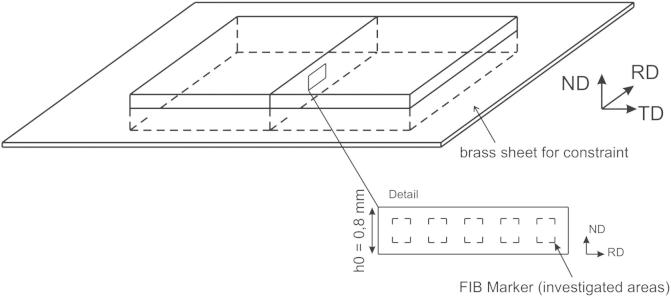
Schematic of the experimental setup used for the quasi in situ EBSD experiments (inset image not to scale).

Throughout this paper, the cold-rolled material will be described using rolling direction (RD), transverse direction (TD) and normal direction (ND), see Fig. 1. In addition to the microstructural and texture investigations, accompanying microhardness measurements with a load of 300 gf were carried out. An overview of the single deformation experiments specifying the geometrical changes and degrees of deformation is given in Table 1.

As later shown, for a specific deformation regime quasi in situ EBSD experiments were carried out.

The principal idea is to obtain OIM data from the same area before and after subsequent rolling steps. To do so, two identical samples were cut out from a copper sheet with a thickness *t *= 0.8 mm, after rolling corresponding to a strain of *ϕ *= 2.1. The ND–RD plane of the two samples was ground and electropolished (Elektrolyte D2 from Struers). A LEO 1540 focused ion beam (FIB) dual beam workstation was used to apply markers in the middle of the sheet height in the ND–RD plane (see schematic drawing in Fig. 1). These markers are suitable to find exactly the same areas after the rolling steps and to ensure that possible drift problems during the EBSD scan are negligible. The height and width of the markers were measured before starting an EBSD scan and compared with the width and height of the markers in the OIM map. To limit the roughness increase along the ND–RD plane during the experiment, which would decrease the quality of the EBSD scans, and to avoid surface effects, the two identically prepared samples were fitted into a brass sheet having a gap that corresponds to the width of the two copper samples. The two polished ND–RD planes were brought into contact with each other and inserted into this gap. Due to the slight expansion of the samples in the TD during rolling, the arising constraint helps the surface roughness to remain negligible and to study the bulk-like behaviour (see Fig. 1). With the described setup the samples (already a *ϕ* = 2.1) and the brass sheet were cold-rolled to a total additional thickness reduction of *ϕ* = 0.7, with reductions of *ϕ* = 0.10–0.15 per pass, in the deformation interval from *ϕ* of 2.1 to 2.8. After each of these rolling increments, the microstructural changes were examined in the FIB marked areas with EBSD scans.

## 3 Results

### 3.1 Microstructural evolution during additional rolling steps after HPT

Microhardness measurements on the as-processed HPT disc showed an almost constant hardness of 142 HV for all radii as the copper disc was deformed to strains that are large enough to reach a steady state. Saturation of the refinement after strains of about *ε* ⩾ 16 were reported in a large number of papers (e.g. [1–9]). The corresponding area-weighted steady-state grain size after HPT processing at room temperature and the given strain rate was found to be 530 nm.

**Fig. 2:**
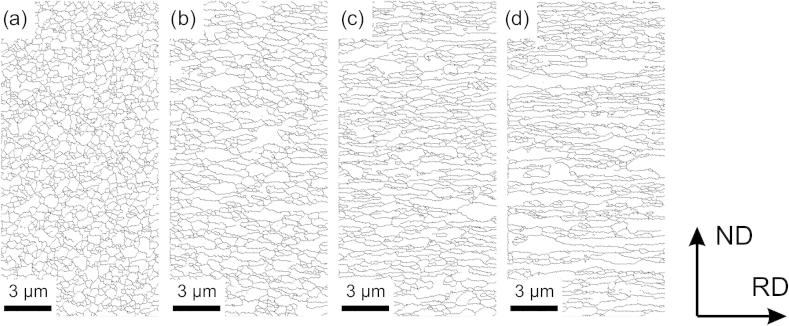
Grain boundary maps showing the evolution of the UFG microstructure at different thickness reductions: (a) after HPT processing, (b) *ϕ* = 0.9, (c) *ϕ* = 2.1, (d) *ϕ* = 5.6.

To study the change of texture and microstructural evolution during additional cold-rolling, EBSD and X-ray diffraction (XRD) measurements as well as microhardness measurements were carried out after various amounts of thickness reduction (see Table 1). Grain boundary maps obtained from OIM data of the microstructures deformed to different thickness reductions can be seen in Fig. 2.

**Fig. 3:**
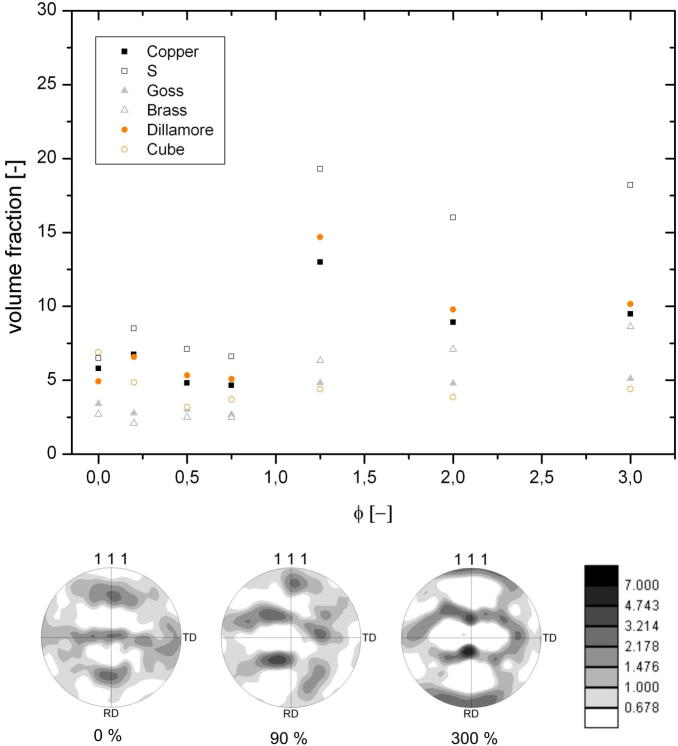
Volume fraction of texture components as a function of the applied thickness reduction *ϕ*. The volume fraction was calculated with a spread of 15°. Three {1 1 1} pole figures show a transition of the shear texture after HPT processing into the typical fcc cold-rolling texture. They were calculated from EBSD data at different thickness reductions.

Fig. 3 shows the volume fraction of individual rolling texture components as a function of the applied thickness reduction, calculated with an allowed spread of 15°. Additionally, {1 1 1} pole figures for the as-HPT-processed material as well as the additionally 90% and 300% cold-rolled material are inserted. From the pole figures it is obvious that texture is changing from a shear texture after HPT processing into the typical cold-rolling texture for copper. From XRD results it can be deduced that texture transition is completed for strains larger than *ϕ* ⩾ 2 where the volume fractions of the individual texture components saturate. At intermediate thickness reductions changes in the volume fraction of the texture components are most pronounced, namely between thickness reductions of about 0.75 ≦ *ϕ* ≦ 1.25, which is consistent with marked hardness changes that will be discussed later.

**Fig. 4:**
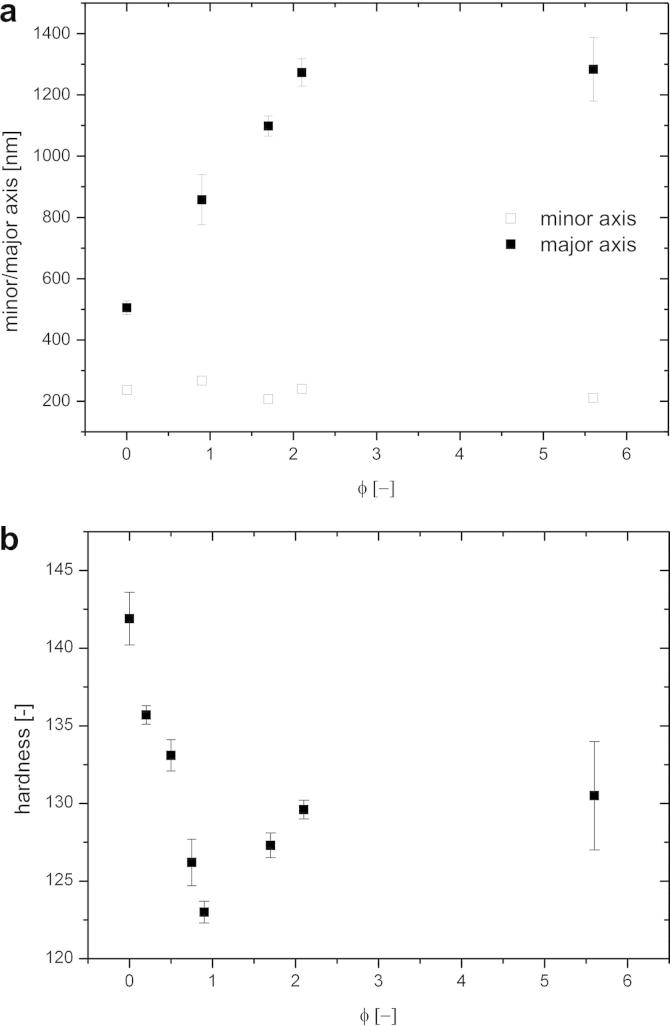
Changes of microstructural parameters and microhardness during cold-rolling: (a) length of the small and large grain axis as a function of thickness reduction; (b) microhardness as a function of *ϕ*.

In addition, microstructural parameters such as grain shape were investigated from EBSD scans. The cold-rolled structure is elongated, thus the area-weighted half axis of the small (minor) and large (major) axis of the grain were calculated as a measure of the grain size. The results of these microstructural investigations are summarized in Fig. 4a. The length of the minor axis changed only negligibly during cold-rolling, see Fig. 4a. The length of the long axis increased during cold-rolling and reached values of ∼1.2 μm at thickness reductions of *ϕ* ⩾ 2. For larger thickness reductions the length of the major axis remained almost constant (Fig. 4a). For calculation of the aspect ratio, the area-weighted length of the minor and major axis of the grains was used. This corresponds to an aspect ratio of ∼5 for thickness reductions larger than *ϕ* ⩾ 2, which is about two times larger in comparison with a HPT processed sample. An examination of the grain size distribution showed that the lengths of the major and minor axes do not exhibit values larger than 4 μm and 0.75 μm, respectively, in the investigated deformation regime. Therefore, elongated grains have to be fragmented again as otherwise the maximum values of the grain axes would change continuously and exceed the aforementioned maximum values.

During cold-rolling the grains become longer but not considerably thinner along their minor axis. This indicates that their mean size has to increase compared to that of the as-processed HPT disc. For the sake of volume consistency it becomes clear that certain grains have to disappear during cold-rolling. In addition to the microstructural investigations, microhardness measurements were carried out on the ND–RD plane at different thickness reductions. Fig. 4b shows the microhardness as a function of the applied thickness reduction *ϕ*. The values measured confirm the above-mentioned results of grain coarsening during cold-rolling at low thickness reductions up to *ϕ* = 0.9, where the lowest hardness of 123 HV was measured. The hardness decrease has a minimum at this thickness reduction, followed by a slight increase again, levelling off for *ϕ* ⩾ 2 at hardness values of 130 HV. This steady-state hardness during rolling is ∼10% lower than the one obtained after HPT processing with 142 HV.

The similarity of the texture and microhardness measurements as well as the evolution of the microstructural parameters show that after thickness reductions of *ϕ* ⩾ 2 no distinct changes with further cold-rolling are observable. In other words, a steady state was restored, which is in contrast to earlier results on severely cold-rolled Ni and Al [18,19]. Surprisingly, the steady-state grain size obtained by cold-rolling is marginally larger than the grain size after HPT, which agrees well with the 10% lower steady-state hardness after cold-rolling.

### 3.2 Quasi in situ experiments

To scrutinize the microstructural changes within the newly found steady-state regime, the aforementioned quasi in situ technique was used. OIMs of six regions of interest, each of them 5 × 11 μm^2^ in size, were continuously observed between subsequent rolling steps within the steady-state regime starting already with *ϕ* = 2.1. The thickness reduction *ϕ* per rolling increment was ∼0.15–0.20. In total, four of these rolling steps were carried out, resulting in an additional thickness reduction of *ϕ* = 0.70, corresponding to a total thickness reduction of *ϕ* = 2.8. Hardness measurements along the ND showed a variation of only 4%. Although such small gradients are present in the sheet, these will not affect any of our findings as always the exactly same area was investigated (centre layer of the sheet).

**Fig. 5:**
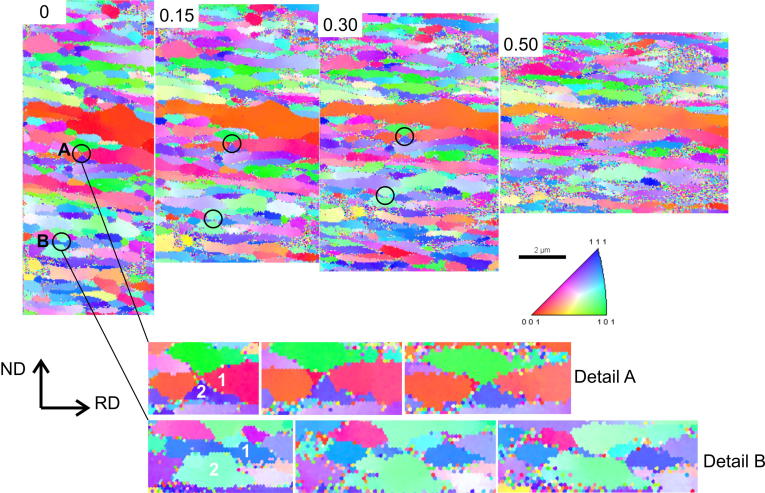
IPF maps showing the microstructural changes during additional thickness reductions of *ϕ* = 0, 0.15, 0.30, 0.50 in the steady-state regime during rolling. Two exemplary positions where characteristic changes are visible are circled and shown in larger magnification for the first three rolling increments at the bottom. Grain 1 represents the shrinking grain and grain 2 the growing grain.

As an example of the measurements, a sequence of inverse pole figure (IPF) maps of one of the investigated areas at additional rolling increments of *ϕ* = 0, 0.15, 0.30 and 0.50 thickness reductions is shown in Fig. 5. No clean-up algorithm was applied, as the scan quality was satisfactory enough and clean-ups generally modify the microstructure, especially in grain boundary areas, which would falsify the results. Errors occurring from drift during the scan can also be neglected for this experiment, as the dimensions of the FIB markings were identical in the IPF maps.

Changes with respect to the microstructure during the additional rolling increments are clearly visible in Fig. 5. Two exemplary positions, labelled A and B, are circled and shown at higher magnifications at the bottom of Fig. 5. From the experiments it can be seen that a sliding of adjacent grains does not occur on the length scale that is accessible with these EBSD scans. If grain boundary sliding was the dominant mechanism on the atomistic level a pronounced shift of adjacent grains after large thickness reductions should be observable, which is not the case. Therefore, at least for the given deformation temperature and strain rate, other mechanisms have to be responsible for the occurrence of the steady-state regime. Quite on the contrary, several grains disappeared during the rolling steps while others grew at their expense. This behaviour is clearly shown in the details of Fig. 5, where a growing grain (grain 2) splits a grain (grain 1 in detail A, B) first, followed by a possible disappearance during further cold-rolling (grain 1 in detail B). This means that the grain boundary of the growing grain moves in the ND. This is surprising as the boundaries aligned in the RD–TD plane are almost planar grain boundaries, thus having no driving force for their movement arising from a reduction in grain boundary energy. Nevertheless, it was shown that shear stresses can lead to the movement of LAGB but also of planar high-angle grain boundaries (HAGBs) [20–22]. A distinct crystallographic relationship between growing and shrinking grains was not observed either, see Fig. 5. As the two details in Fig. 5 show, the growing and shrinking grains do not necessarily need to have a similar orientation, see Fig. 5, detail A.

## 4 Discussion

The results of the present study raise several questions that will be discussed within this section. First of all, it should be discussed in more detail what mechanisms enable the steady-state deformation with a constant average grain size. Secondly, the coarsening of the grains, especially at low rolling strains, during the transition of the UFG structure after HPT processing into the cold-rolled UFG microstructure will be treated, which finally leads to a larger grain size after cold-rolling compared to HPT processing at the same temperature.

### 4.1 Mechanisms enabling a steady state

**Fig. 6:**
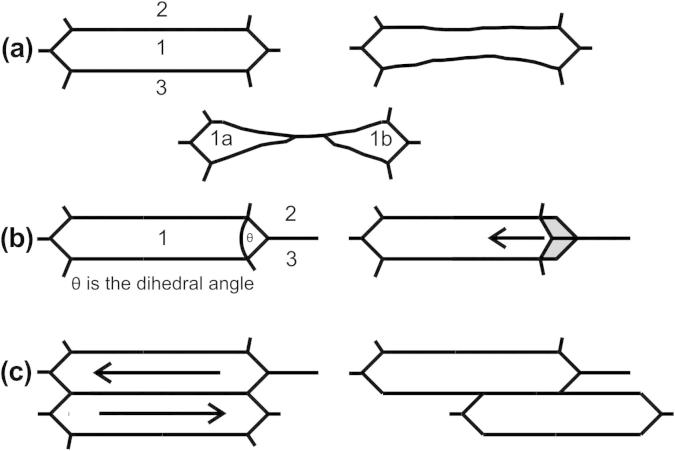
Schematic of possible restoration mechanisms enabling a steady state: (a) grain boundary motion; (b) triple junction motion; (c) slide event of adjacent grains. Grain 1 represents the shrinking grain, 2 and 3 the growing grains and 1a and 1b are the fragments of grain 1.

The occurrence of a steady state during plastic deformation makes restoration mechanisms necessary that, on average, compensate for the refinement caused by additional deformation. Potential mechanisms are schematically shown in Fig. 6. Such mechanisms could be the movement of grain boundaries leading to the disappearance of certain grains, while others grow at their expense (Figs. 5 and 6a); the movement of triple junctions (Fig. 6b), which would also enable the growth of neighbouring grains accompanied by a reduction of the aspect ratio; or the sliding of adjacent grains (Fig. 6c). From the experiments carried out, grain boundary migration along the ND was found to be the dominant restoration mechanism (see Fig. 5). Therefore, the necessary boundary velocity to balance the refinement caused by plastic deformation should be estimated.

For the experimental conditions with an average thickness reduction of *ϕ* = 0.15 per pass (*ε* = 0.17) and an average steady-state grain size (boundary spacing) in the ND of 440 nm a refinement of 61 nm per pass can be calculated. In other words, each boundary has to move 30 nm in ND for a strain increment of *ε *= 0.17 or ∼180 nm for a strain of *ε *= 1 to balance the caused refinement. With a strain rate of $\overset{˙}{\varepsilon }=10^{-1}\;{\text{s}}^{-1}$ and the mentioned strain increment of *ε *= 0.17 per pass, the time interval Δ*t* for plastic deformation is ∼1.7 s and thus the necessary average grain boundary velocity, *v*, yields to approximately *v* ∼ 18 nm s^−1^. This seems to be quite a high value for grain boundary migration at room temperature. Nevertheless, grain boundary velocities in the same order of magnitude were experimentally measured in aluminium bicrystals [20]. These relatively high values were attributed to stress driven grain boundary motion. Therefore, the steady-state grain size seems to be a consequence of a dynamic equilibrium of grain refinement and mechanically induced grain boundary migration, which was suggested also in [1]. This equilibrium condition must be fulfilled for the entire deformed volume and not only for a single grain. In addition to the mechanically induced boundary migration, triple junction motion was mentioned of being a potential restoration mechanism (Fig. 6b) and has recently been identified as a recovery mechanism in severely cold-rolled aluminium [23,24]. The driving force for triple junction motion increases if the dihedral angle *θ*, as shown in Fig. 6b, decreases. However, triple junction motion can neither be excluded nor confirmed by the experiments carried out in this study as the disappearance of a grain after being split by a migrating boundary could be realized either by further movement of the grain boundary, by triple junction motion, or through a combination of both. With the used experimental technique both possible mechanisms cannot be separated. Results of the mentioned recent studies showed that triple junction motion was not frequently observed at ambient temperature as it is a thermally activated process [23,24]. Therefore, it is reasonable that their contribution to the restoration of a microstructure within the steady state at low homologous temperatures should not be dominant. Exceptions might occur when grains become thin during deformation, which is also accompanied by a decrease in the dihedral angle *θ* or at higher homologous deformation temperatures. A more frequent movement of triple junctions and a higher mobility of the grain boundaries at elevated temperature would also explain the observed smaller grain aspect ratio and larger steady-state grain size at higher homologous deformation temperatures [1].

As mentioned before, no preferred crystallographic relation was observed between shrinking and growing grains. This local observation is supported by the fact that the volume fractions of the individual crystallographic texture components do not change within the steady state, see Fig. 3.

It is of great importance to think of the driving forces responsible for the movement of the grain boundaries. In principle, UFG or nc structures possess high latent energy due to their large fraction of grain boundaries. An estimation of the energy stored in HAGB yields values of the total grain boundary energy, *E_gb_*, of several MJ cm^−3^. As a consequence of these high values, a reduction of the grain boundary energy seems likely to be the driving force for the grain boundary movement. A closer inspection, however, shows that this cannot be responsible for the typical rolling structure that consists of a very large grain boundary area parallel to the RD in this study. No grain boundary energy can be saved if an almost planar boundary is moving. It is even enlarged when a shrinking grain is fragmented, see Fig. 6a, because energy would be only saved when the shrinking grain completely disappears.

Recent results suggest that mechanical stresses seem to be important for the movement of (planar) boundaries and often nc or UFG structures showed drastic coarsening when subjected to high stress levels [22,25–27]. In view of these results combined with our findings, the driving force for the observed grain boundary movement seems to be a result of local stress differences between adjacent grains and not necessarily the high stress levels itself. These stress differences can originate from differences in grain size or Taylor factor. The stress differences yield a difference in elastic strain energy stored during deformation and a difference in the plastic work to be spent to accommodate the external applied strain. In contrast to the grain boundary energy, plastic work or elastic strain energy can also be saved when only a small part of the boundary is moving while the shrinking grain becomes fragmented. Under the assumption of a linear elastic and ideal plastic behaviour of the material that is well fulfilled in UFG metals, the plastic work for deformation scales linearly with the stress. Larger driving forces would result from elastic strain energies, where the energy is proportional to the stress level squared.

A simple estimation of the maximum elastic strain energy that can be saved by the movement of a boundary can be performed as follows. Maximum stress differences will arise when a large and a small grain are next neighbours. For simplicity the two grains were regarded as cuboids of 100 nm and 500 nm in thickness, respectively (minor axis), 1000 nm in length (major axis) and 500 nm in width. The values were taken with respect to microstructural measurements as shown in Fig. 4 and the minimum and maximum values obtained from the distribution of the length of the minor axis. The width was estimated to be the average length measured from OIM recorded along the ND–TD plane. The yield stress of the 500 nm thick grain was taken from literature, having a value of ∼440 MPa [28]. Assuming a Hall–Petch type relationship (with an exponent of −0.5) the yield stress of a 100 nm grain can be estimated to be in the order of 800 MPa. This value is in relatively good agreement with the literature [29]. With these assumptions one can calculate the difference in elastic strain energy density to be ∼2 MJ m^−3^, which corresponds with a driving pressure on the boundary of 2 MPa. Literature values for the driving pressures during recrystallization of cold-worked metals are within this range, namely 2–20 MPa [30]. The activation energies for the migration of a HAGB are ∼10^−19^ J or 1 eV per atom. For hot-working or static recrystallization processes it seems clear that this movement is thermally activated. This is not the case for deformation at room temperature and so it is reasonable to assume that plasticity is necessary to overcome this activation barrier. Despite the clarity of this simple estimation, the assumption will bear further scrutiny with suitable experiments.

**Fig. 7:**
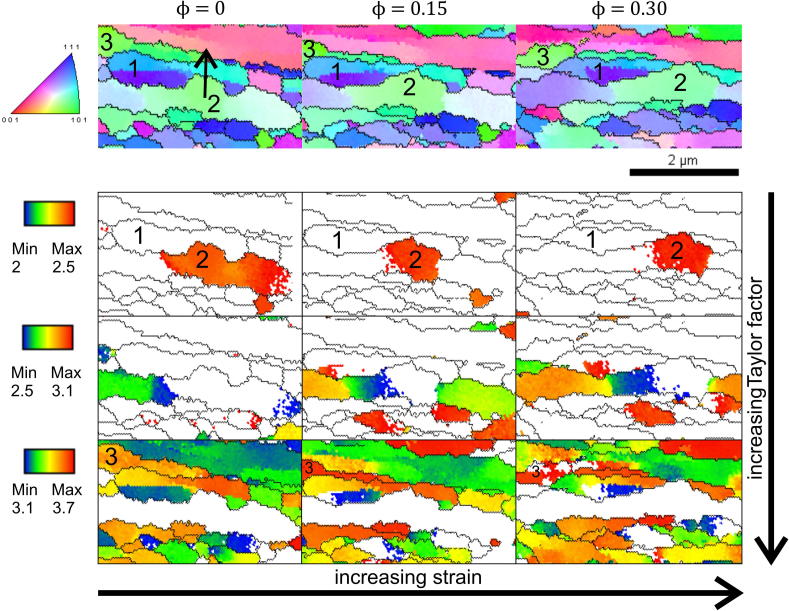
Possible driving force for grain growth. The IPF maps of a region where a grain shrinks and fragments (grain 1) during additional rolling are shown. The Taylor factors are plotted beneath at different scales for a better visualization of the differences. The growing grain (grain 2) shows considerably lower Taylor factors than the fragmented (grain 1) one.

As previously mentioned, the local stress differences between two adjacent grains could also arise from differences in the Taylor factor. To analyze this, careful investigation of the IPF maps of the quasi in situ experiment in combination with the Taylor factors calculated for rolling were conducted. The results are shown in Fig. 7. In the IPF maps at the top of Fig. 7 it can be seen that grain 1 fragments as grain 2 grows. The direction of the grain boundary movement is indicated with an arrow. Taylor factor maps are plotted underneath, encoded with colours in a rather small factor interval to better visualize their differences. It can be seen that the fragmented grain (grain 1) has a Taylor factor 50% larger than the growing grain (grain 2). The same is true for shrinking grain 3, which has a larger Taylor factor than the adjacent grain. Also, for most of the other positions where grains disappeared, stress difference due to different Taylor factors and/or different grain sizes appeared.

It should further be mentioned that growing grains could also shrink again as, due to crystal rotation (see Fig. 5), their Taylor factor is changing during deformation. Additionally, grains could also get into contact with an adjacent grain of similar size or similar Taylor factor, which would also minimize the driving force and lead to refinement again. This is indeed necessary to obtain a steady-state grain size distribution and to ensure that grains do not become arbitrarily large. In Ref. [1] it was suggested that larger grains become subdivided again through the increase of LAGBs having a high-angle grain boundary character with increasing strain. However, such a mechanism has not been observed here. On the contrary, it seems that grain boundary motion itself limits the maximum length of the grains.

### 4.2 Structural transition and steady state after cold-rolling

**Fig. 8:**
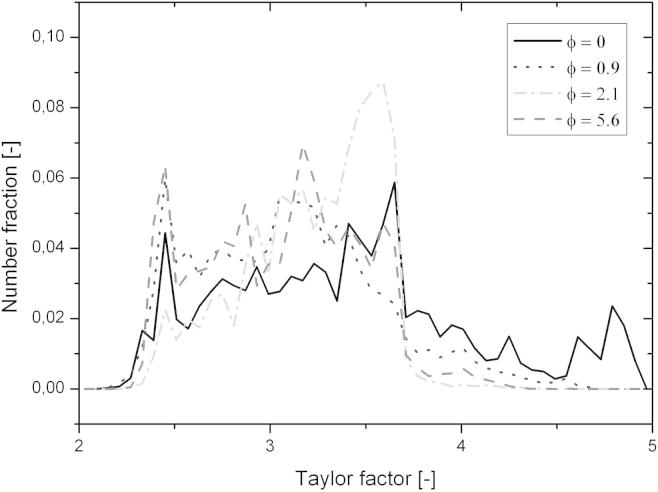
Distribution of the Taylor factors calculated for cold-rolling for microstructures at different thickness reductions.

Studying the microstructural transition from HPT-processed copper to the steady-state structure after cold-rolling revealed that a pronounced coarsening up to thickness reductions of *ϕ* = 0.9 took place, see Fig. 4. The accompanying drop in hardness was followed by a slight increase. The steady-state hardness had a value of 130 HV, which is ∼10% lower than the corresponding steady-state value after HPT processing (see Fig. 4b). The observed behaviour during this structural transition that finally leads to the steady state discussed in the previous section can be explained as follows. Texture components have to change from a shear texture after HPT processing into the face centred cubic (fcc) rolling texture. Furthermore, the volume fraction of certain texture components has to increase (see Fig. 3). This process is completed with the onset of the steady state after *ϕ* ⩾ 2. As the same texture occurred for cold-rolled coarse grained fcc materials [31], one can conclude that plastic deformation is still dislocation-mediated. A similar behaviour was also observed for cold-rolled UFG copper processed by equal channel angular pressing, although smaller thickness reductions were applied [32]. In principle, the texture change can be realized by either crystal rotation and/or shrinkage of grains having an unfavourable orientation for rolling. As coarsening was observed during the initial stage of cold-rolling and the minimum hardness measured corresponds well with the formation of rolling texture components (see Figs. 3 and 4b), the latter proposed mechanism seems to be the dominant one although lattice rotation cannot be excluded. This hypothesis is confirmed by comparing the distribution of the Taylor factor of microstructures deformed to different thickness reductions (see Fig. 8). From Fig. 8 it is clearly visible that the UFG structure obtained by HPT is not favourably oriented for cold-rolling as ∼20% of the grains have a Taylor factor larger than 3.7. An increasing thickness reduction leads to a decrease in the number fraction of less favourably oriented grains. It has been stated that the driving force for the observed grain boundary motion arises from local stress differences due to different grain sizes or Taylor factors. Regarding the above-mentioned results the latter one seems to be dominant up to thickness reductions *ϕ* < 1; however, the maximum possible difference in Taylor factor between grains diminishes with increasing strain (see Fig. 8). This in turn leads to smaller driving forces for the grain boundary movement with increasing thickness reduction. As a consequence the refinement per strain increment is larger than the restoration rate, resulting in a net refinement of the structure until an equilibrium condition between these two competing processes is reached. This explanation is in good agreement with the experimental results (see Figs. 4b and 8). Despite this, the 10% lower steady-state hardness value after cold-rolling is not satisfactorily explained above.

A strain rate difference between HPT and cold-rolling could principally explain the different steady-state grain size [1,4]. The strain rate during cold-rolling $\left(\overset{˙}{\varepsilon }=10^{-1}\;{\text{s}}^{-1}\right)$ was, however, larger than in the case of HPT $\left(\overset{˙}{\varepsilon }=10^{-3}\;{\text{s}}^{-1}\right)$, which contradicts this simple explanation. Effects resulting from heating due to plastic deformation are negligible for both processes as well, because the strain rates were rather small. Additionally, more plastic work has to be spent for realizing a certain strain when the sample is deformed by simple shear instead of rolling, and so one could expect even a smaller grain size for cold-rolling [33].

The considerations of a dynamic equilibrium between restoration and refinement outlined in Section 4.1 lead to the conclusion that in the case of HPT deformation the velocity of the boundaries must be lower than during cold-rolling, leading to a smaller amount of growth per strain increment accompanied by a smaller steady-state grain size in the case of HPT. One reason for that might be the absence of a large hydrostatic pressure during rolling. There is experimental evidence that hydrostatic pressure impedes the grain boundary mobility [34] and similar effects on the steady-state grain size were also reported for copper processed by HPT with different amounts of applied pressure [2,35]. Thus, it is reasonable that for copper a contribution to the enhanced grain boundary mobility during rolling might be due to the absence of hydrostatic pressure.

## 5 Conclusion

In the present work UFG copper was processed by HPT and then cold-rolled to strains until another steady state was reached. Afterwards, suitable quasi in situ experiments were carried out to reveal the restoration mechanisms necessary to obtain this new steady state. The main results can be summarized as follows:

1. Grain boundary motion along the ND is the dominant restoration mechanism in the steady state regime during cold-rolling.
2. Stress differences between adjacent grains appear to be the driving force for the observed grain boundary migration.
3. The deformation process itself has an influence on the mobility of grain boundaries and as a consequence affects the achievable steady-state grain size and hardness.

## Figures and Tables

**Table 1:** Overview of the investigations carried out on samples deformed to different thickness reductions with *h* the height of the copper sheets, *ϕ* the logarithmic thickness reduction, *ε_CR_* the strain applied by rolling and *ε_tot_* the total strain.

*h* (mm)	*ϕ*	*ε_CR_*	*ε_tot_*
6.5	0	0	92.1^a^
2.68	−0.90	1.00	93.1
1.19	−1.70	2.00	94.1
0.80	−2.10	2.40	94.5
0.35	−2.90	3.40	95.5
0.025	−5.60	6.40	98.5
0.007	−6.80	7.90	100.0

a Equivalent von Mises strain of HPT deformation calculated for a radius *r* = 11 mm.

## References

[1] Pippan R., Scheriau S., Taylor A., Hafok M., Hohenwarter A., Bachmaier A. (2010). Annu Rev Mater Res.

[2] Hebesberger T., Stüwe H.P., Vorhauer A., Wetscher F., Pippan R. (2005). Acta Mater.

[3] Valiev R.Z., Ivanisenko Yu.V., Rauch E.F., Baudelet B. (1996). Acta Mater.

[4] Vorhauer A., Pippan R. (2008). Metall Mater Trans A.

[5] Sabirov I., Perez-Prado M.T., Molina-Aldareguia J.M., Semenova I.P., Salimgareeva G.K., Valiev R.Z. (2011). Scr Mater.

[6] Edalati K., Toh S., Ikoma Y., Horita Z. (2011). Scr Mater.

[7] Popov V.V., Popova E.N., Stolbovskiy A.V., Pilyugin V.P. (2011). Mater Sci Eng A.

[8] Pippan R., Wetscher F., Hafok M., Vorhauer A., Sabirov I. (2006). Adv Eng Mater.

[9] Tsuji N., Kamikawa N., Li B. (2007). Mater Sci Forum.

[10] Yang B., Vehoff H., Hohenwarter A., Hafok M., Pippan R. (2008). Scr Mater.

[11] Zhilyaev A.P., Swaminathan S.C., Pshenichnyuk A.I., Langdon T.G., McNelley T.R. (2013). J Mater Sci.

[12] Divinski S.V., Padmanabhan K.A., Wilde G. (2011). Philos Mag.

[13] Edalati K., Horita Z. (2011). Scr Mater.

[14] Mohamed F.A. (2001). Acta Mater.

[15] Zhao Y.H., Liao X.Z., Zhu Y.T., Horita Z., Langdon T.G. (2005). Mater Sci Eng A.

[16] Hohenwarter A., Bachmaier A., Gludovatz B., Scheriau S., Pippan R. (2009). Int J Mater Res.

[17] Fritz A., Schulze G. (2010). Fertigungstechnik.

[18] Hughes D.A., Hansen N. (2000). Acta Mater.

[19] Liu Q., Huang X., Lloyd D.J., Hansen N. (2002). Acta Mater.

[20] Winning M., Gottstein G., Shvindlerman L.S. (2001). Acta Mater.

[21] Winning M., Gottstein G., Shvindlerman L.S. (2002). Acta Mater.

[22] Rupert T.J., Gianola D.S., Gan Y., Hemker K.J. (2009). Science.

[23] Yu T., Hansen N., Huang X. (2013). Proc R Soc A.

[24] Yu T., Hansen N., Huang X. (2012). Philos Mag.

[25] Agnew S.R., Weertman J.R. (1998). Mater Sci Eng A.

[26] Höppel H.W., Zhou Z.M., Mughrabi H., Valiev R.Z. (2002). Philos Mag.

[27] Zhang K., Weertman J.R., Eastman J.A. (2004). Appl Phys Lett.

[28] Hohenwarter A., Pippan R. (2012). Mater Sci Eng A.

[29] Wang Y.M., Wang K., Pan D., Lu K., Hemker K.J., Ma E. (2003). Scr Mater.

[30] Stüwe HP. In: Haessner F, editor. Recrystallisation of metallic materials. Stuttgart: Dr. Riederer Verlag; 1978.

[31] Hirsch J., Lücke K. (1988). Acta Metall.

[32] Mishin O.V., Gottstein G. (1998). Philos Mag.

[33] Stüwe H.P. (2003). Adv Eng Mater.

[34] Hahn H., Gleiter H. (1979). Scr Metall Mater.

[35] Hebesberger T., Pippan R., Stüwe H.P., Zhu Y.T. (2002). Ultrafine grained materials II.

